# Focused extracorporeal shock wave therapy as a salvage option for humeral shaft delayed union and non-union with implant loosening or fixation-related concerns: a report of three cases

**DOI:** 10.1016/j.xrrt.2026.100826

**Published:** 2026-07-22

**Authors:** Jinyoung Jeong, Jae-Ran Seol, Seo-Hee Choi, Seheon Lee

**Affiliations:** Department of Orthopaedic Surgery, St. Vincent's Hospital, The Catholic University of Korea, Suwon, Republic of Korea

## Introduction

Delayed union and non-union of humeral shaft fractures remain challenging clinical problems, particularly when delayed healing is accompanied by implant loosening or fixation-related concerns. Stable fixation is a fundamental principle of fracture healing, and revision fixation with or without bone grafting is generally considered the standard treatment for established humeral shaft non-union or implant failure. Common revision strategies include compression plating, double plating when appropriate, plate-and-bone-strut constructs, and biological or artificial bone graft augmentation, depending on the anatomic level of the humerus, bone quality, previous fixation, and residual stability.^1^^,^^8^, ^9^, ^10^, ^11^ However, revision surgery may require additional soft-tissue dissection, increase the risk of neurologic irritation or injury, and may not always be acceptable to the patient.

Extracorporeal shock wave therapy (ESWT) has been reported as a non-invasive method for stimulating bone healing in selected long-bone delayed union and non-union cases.^2^^,^^4^^,^^5^^,^^12^, ^13^, ^14^ International Society for Medical Shockwave Treatment guidance recognizes delayed fracture healing and pseudarthrosis/non-union as bone-related indications for ESWT after appropriate clinical and imaging assessment, while emphasizing expert indication and avoidance of contraindicated target tissues such as the lung, brain or spinal cord, tumor tissue, epiphyseal regions, and clinically relevant coagulopathy.^6^ Because ESWT primarily provides biological stimulation rather than mechanical stabilization, its role in the presence of implant loosening or fixation-related mechanical compromise remains controversial. Grossly unstable non-union, progressive displacement, or implant breakage generally requires surgical stabilization.

Herein, we report 3 cases of humeral shaft delayed union or non-union with implant loosening or fixation-related concerns treated with focused ESWT. We hypothesized that, in carefully selected patients with residual construct stability and without catastrophic fixation failure, focused ESWT would be followed by renewed callus progression and eventual union, while not replacing revision fixation for clearly unstable non-union. This report highlights the potential salvage role and technical considerations of focused ESWT in selected patients with mechanical compromise short of catastrophic construct failure, for whom revision fixation would ordinarily be considered but was refused or clinically undesirable.

## Case report

This is a retrospective case report of 3 completed cases treated between 2019 and 2024 at a tertiary university hospital. Case 1 underwent the index operation at another hospital and was subsequently referred to our institution; Cases 2 and 3 underwent the index fixation procedures at our institution by a single fellowship-trained shoulder surgeon. The study was approved by the institutional review board of St. Vincent's Hospital, the Catholic University of Korea (IRB No. VC25ZISI0261). Written informed consent for publication of clinical information and radiographic images was obtained from all patients.

Early delayed union was considered when radiographs showed insufficient progression of fracture healing at approximately 3 to 4 months after fixation. Established delayed union was considered when insufficient radiographic progression persisted beyond 6 months. Late-stage non-union was considered when lack of progressive healing persisted beyond 12 months, particularly in association with implant loosening. Radiographic union was defined as progressive bridging callus or cortical continuity on serial orthogonal radiographs with clinical improvement at the fracture site. The diagnosis and assessment of healing were based on serial orthogonal plain radiographs and focal clinical findings, including fracture-site tenderness and pain with arm use; computed tomography was not routinely obtained. Infection was assessed clinically and with laboratory testing, including inflammatory markers when clinically indicated, and no patient had findings suggestive of infection before ESWT. Implant loosening and fixation-related mechanical compromise were judged on serial radiographs rather than by a quantitative loosening score or mechanical testing. Baseline smoking status, alcohol abuse, diabetes, and major comorbidities were reviewed. Bone mineral density testing and a formal metabolic bone workup were not routinely performed. Formal visual analog scale pain scores, range-of-motion values, and validated upper-extremity outcome scores were not available for these retrospective cases.

In our practice, fixation-related mechanical compromise was not considered an absolute contraindication to ESWT when the construct still provided residual functional stability. Limited screw backing-out or implant loosening without progressive displacement, implant breakage, or a clinically flail painful segment was considered potentially suitable for ESWT with close radiographic monitoring. In contrast, patients with catastrophic construct failure, progressive displacement, implant breakage, active infection, or an unsafe acoustic pathway were not considered appropriate candidates for ESWT alone.

**Figure 1:**
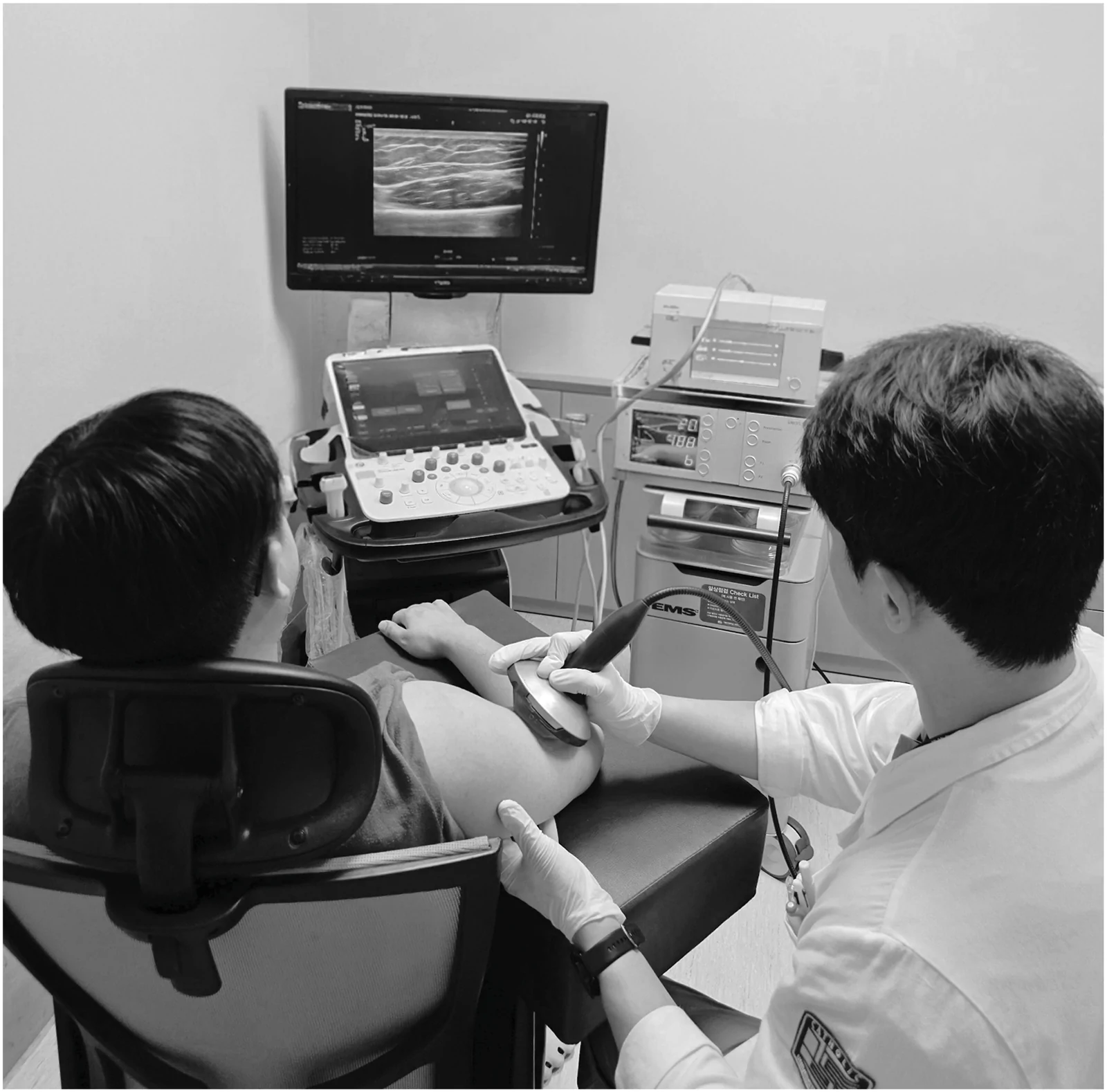
Representative black-and-white photograph demonstrating focused ESWT setup using a volunteer model. The subject is seated, with the modeled treatment arm supported on a pillow on the treatment table. After ultrasound localization of the humeral shaft target region, the focused ESWT applicator is positioned over the sonographically identified target area. This setup helps maintain a stable acoustic window, minimize painful arm rotation, and avoid the thoracic cavity and major neurovascular structures. This photograph is intended only to illustrate the treatment setup and does not show a study patient. *ESWT*, extracorporeal shock wave therapy.

All treatments were performed using a piezoelectric-type focused ESWT device under sonographic guidance. The treatment protocol was defined by the corresponding author according to institutional practice and ISMST-based principles. All ESWT sessions were implemented at the same tertiary university hospital and were delivered by an orthopaedic surgeon with ISMST certification. For humeral shaft lesions, patients were treated in a sitting position, with the affected arm resting on a pillow on the treatment table to minimize painful rotation and expose the humeral shaft. The fracture or non-union interface was first localized with ultrasound, and the focused ESWT applicator was then positioned over the sonographically identified target region through a safe acoustic window. A representative clinical photograph using a volunteer model was added to demonstrate the sitting position, pillow-supported arm, ultrasound localization, and focused ESWT applicator placement (Fig. 1). The treatment focus was directed to the radiographic and sonographic fracture gap, non-union interface, and surrounding callus margins, while avoiding adjacent major neurovascular structures and, when applicable, the thoracic cavity. After treatment, patients were advised to use an arm sling for comfort and to avoid painful lifting, torsional stress, or forceful arm use until radiographic progression was observed; gentle pain-limited shoulder, elbow, wrist, and hand motion was permitted.

### Case 1. Late-stage non-union with intramedullary nail loosening

A 49-year-old woman sustained a right humeral shaft fracture in a traffic accident and underwent intramedullary nailing at another hospital. She was referred because of persistent pain, symptoms suggestive of radial nerve involvement, implant loosening, and delayed fracture healing. Electrodiagnostic testing confirmed radial nerve injury, and the neurologic symptoms improved gradually over approximately 6 months after referral. She was a non-smoker, had no history of alcohol abuse, and did not have diabetes mellitus. Laboratory findings did not suggest infection, and there was no clinical evidence of infection.

Serial radiographs obtained at 5 and 12 months after the index operation demonstrated persistent non-union with minimal callus formation and loosening of the intramedullary nail. On examination, she had focal tenderness at the non-union site and pain with shoulder internal and external rotation, with a subjective sensation of motion at the fracture site; however, there was no progressive displacement, implant breakage, or gross flail segment. Because this represented substantial mechanical compromise, revision fixation with bone grafting was recommended as the standard treatment option. However, the patient declined further surgery. Focused ESWT was therefore selected as a salvage option after discussion with the patient. ESWT was applied to the non-union interface and callus margins at an energy flux density of 0.4 mJ/mm^2^, with 3,000 impulses per session, for a total of 4 sessions.

**Figure 2:**
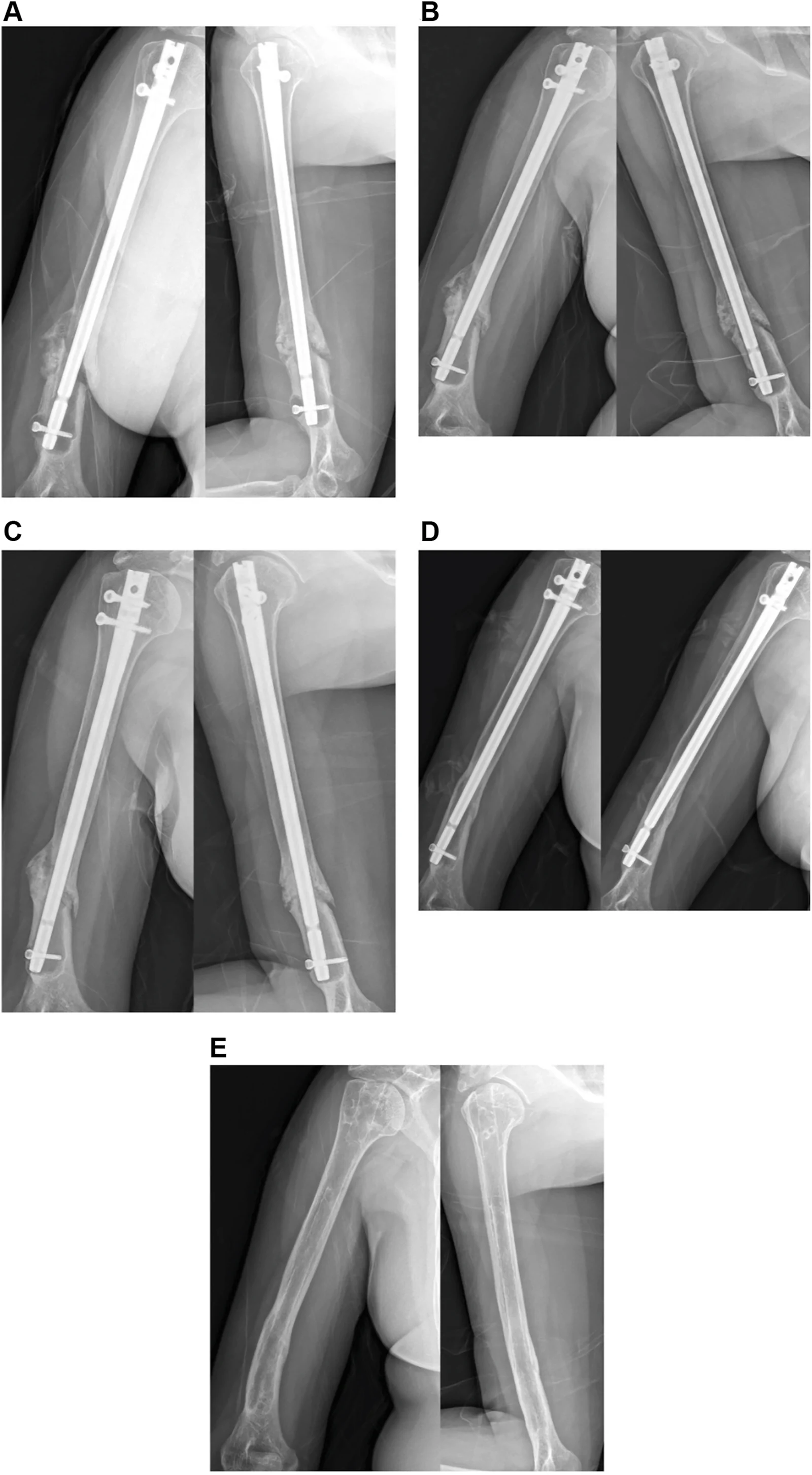
Serial radiographs of Case 1, a 49-year-old woman with late-stage non-union and intramedullary nail loosening after fixation for a right humeral shaft fracture. (**A**) Persistent non-union with implant loosening at 12 months post-operatively. (**B** and **C**) Progressive callus formation at 2 and 8 months after extracorporeal shock wave therapy (ESWT). (**D**) Further consolidation at 24 months after ESWT. (**E**) Radiograph after implant removal showing maintained alignment and union.

After ESWT, progressive radiographic callus formation was observed. Early healing was noted at 2 months after treatment, substantial consolidation was evident within 8 months, and remodeling was maintained through the 24-month follow-up. The radial nerve symptoms improved spontaneously without additional intervention, and ESWT did not aggravate the pre-existing neurologic symptoms. After confirmation of solid union, the intramedullary nail was removed, and final radiographs demonstrated maintained alignment and remodeling of the humeral shaft (Fig. 2).

### Case 2. Established delayed union after plate fixation with fixation-related concerns

A 60-year-old man sustained a left humeral shaft fracture after a fall and underwent open reduction and internal fixation with a locking compression plate at our institution by the same fellowship-trained shoulder surgeon. He was a non-smoker, had no history of alcohol abuse, and had controlled diabetes mellitus. There was no evidence of infection on clinical evaluation or laboratory testing.

Follow-up radiographs at 6 and 12 months post-operatively demonstrated persistent delayed union with insufficient callus formation and radiographic plate-related mechanical compromise, including limited screw/implant loosening. He had persistent arm pain and focal tenderness at the delayed-union site, but the humerus moved as a single functional unit during gentle examination, and there was no progressive displacement, plate breakage, or global loss of fixation that mandated immediate revision fixation. Revision fixation with or without bone grafting was discussed because stable fixation is generally considered essential for fracture union; however, non-operative treatment with focused ESWT was chosen after discussion with the patient. ESWT was performed under sonographic guidance, targeting the delayed-union interface and adjacent callus margins, at an energy flux density of 0.4 mJ/mm^2^, with 4,000 impulses per session. Three sessions were administered at 2-week intervals.

**Figure 3:**
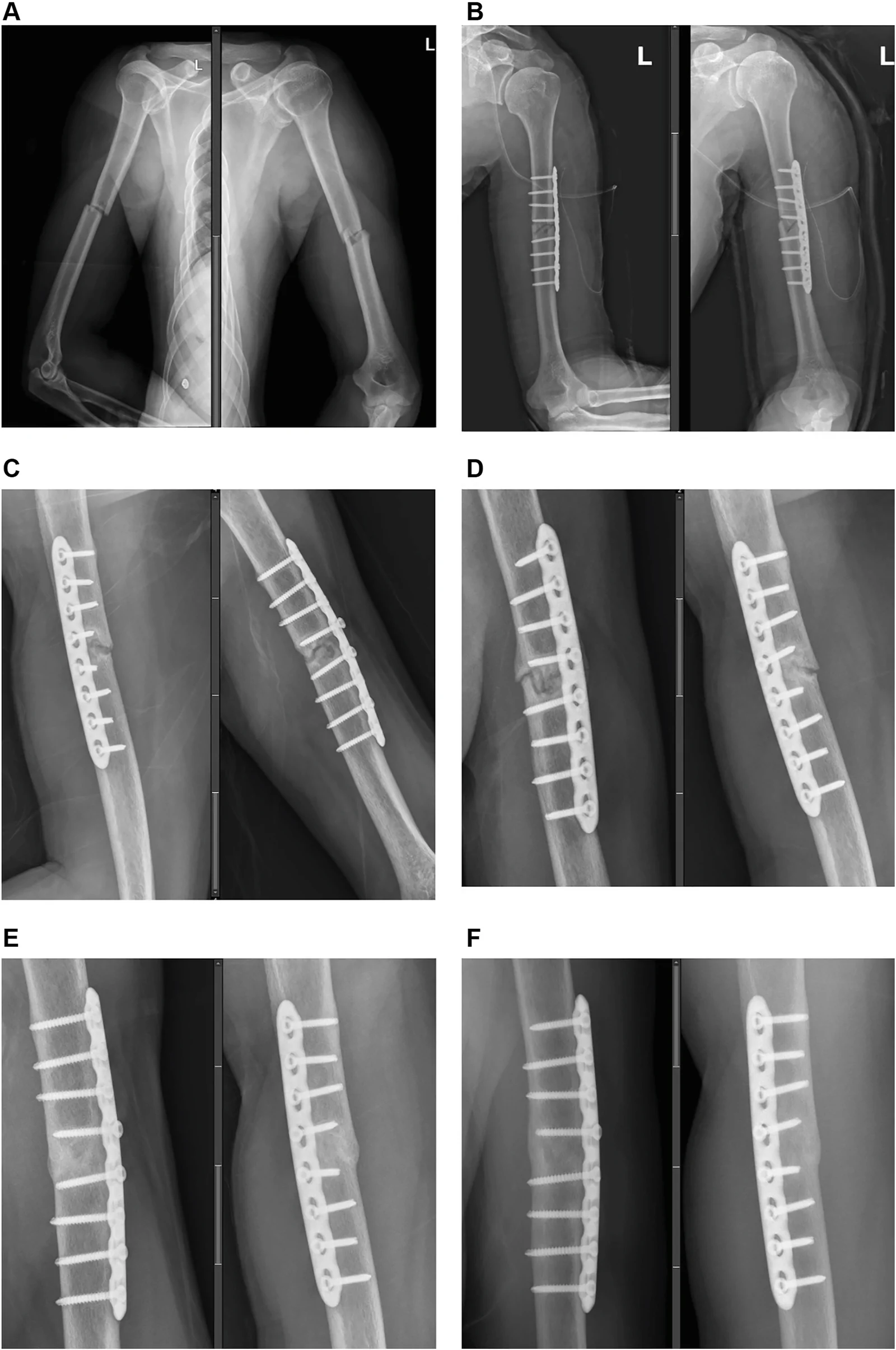
Serial radiographs of Case 2, a 60-year-old man with established delayed union and plate-related mechanical compromise after plate fixation for a left humeral shaft fracture. (**A**) Initial fracture radiograph. (**B**) Immediate post-operative radiograph after plate fixation. (**C**) Persistent delayed union at 12 months post-operatively. (**D**) Radiograph obtained 1 month after the third ESWT session showing early callus formation. (**E**) Progressive consolidation at 12 months after the third ESWT session. (**F**) Maintained union at the 2-year follow-up. *ESWT*, extracorporeal shock wave therapy.

Radiographs obtained after the second and third ESWT sessions showed gradual callus formation. Further progression of bone healing was observed at 7 and 12 months after the third ESWT session, and union was maintained at the 2-year follow-up. The patient reported gradual symptom improvement, and no treatment-related complications occurred (Fig. 3).

### Case 3. Early delayed union after plate fixation with concern for screw backing-out

A 72-year-old man sustained a right humeral shaft fracture after a fall and underwent open reduction and internal fixation with a locking compression plate at our institution by the same fellowship-trained shoulder surgeon. He was a non-smoker, had no history of alcohol abuse, and did not have diabetes mellitus. No evidence of infection was identified clinically or on laboratory testing.

At 3.5 months post-operatively, serial radiographs demonstrated insufficient callus formation and delayed progression of fracture healing, with concern for early backing-out of the interfragmentary screw. The patient had activity-related arm pain and tenderness around the fracture region, but the humerus moved as a single functional unit and there was no gross displacement, plate breakage, or catastrophic loss of fixation. Although this time point was early for established delayed union or non-union, the combination of insufficient callus formation and screw backing-out was considered an early warning sign for possible progression to fixation failure. Focused ESWT was therefore initiated as an early adjunctive biological stimulation under sonographic guidance, targeting the fracture line and surrounding callus-forming region. ESWT was applied at an energy flux density of 0.4 mJ/mm^2^, with 4,000 impulses per session. Three sessions were administered at 6- to 8-week intervals.

**Figure 4:**
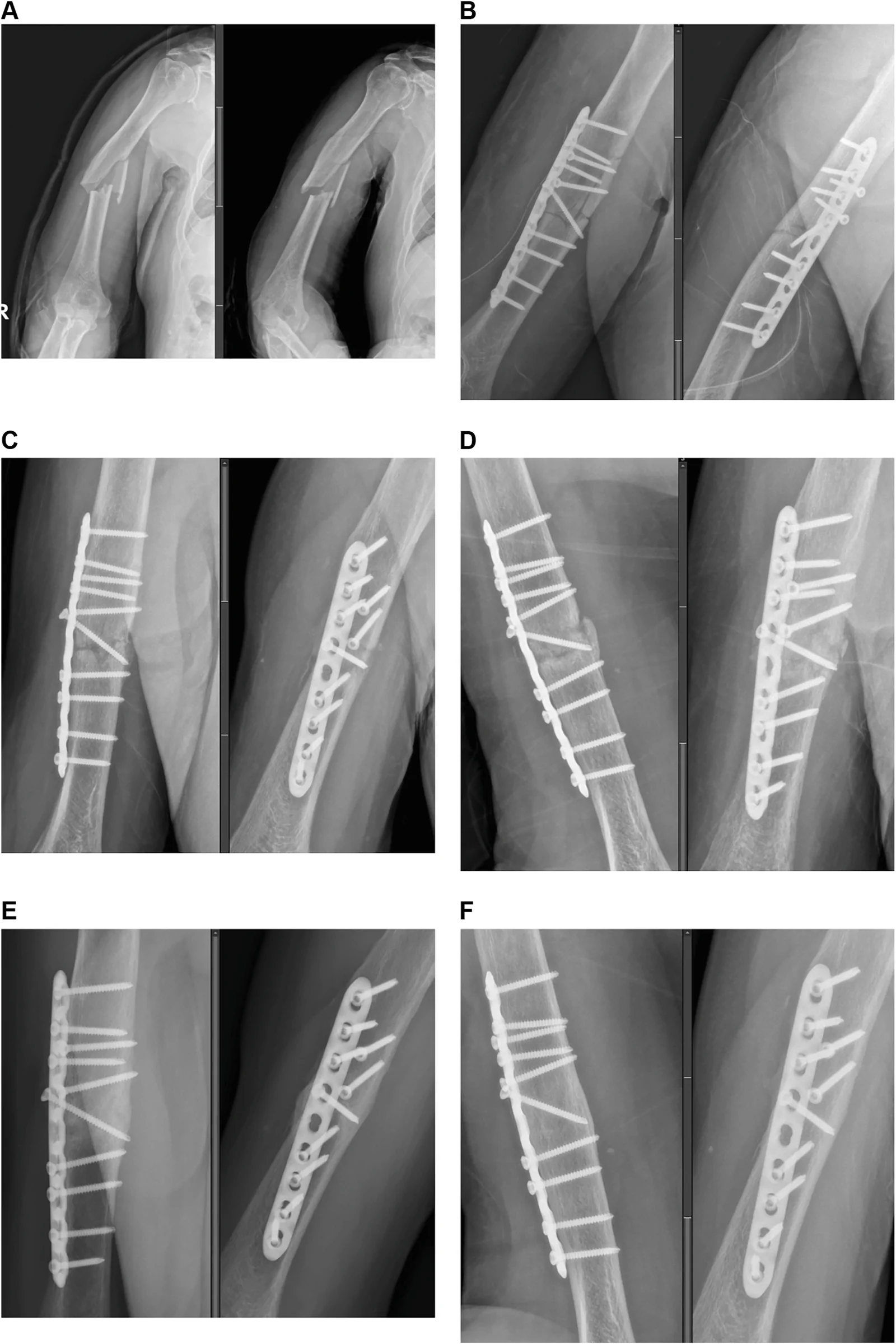
Serial radiographs of Case 3, a 72-year-old man with early delayed union and concern for interfragmentary screw backing-out after plate fixation for a right humeral shaft fracture. (**A**) Initial fracture radiograph. (**B**) Immediate post-operative radiograph after plate fixation. (**C**) Early delayed union at 3.5 months post-operatively with concern for screw backing-out. (**D**) Radiograph after the second ESWT session showing callus formation. (**E**) Progressive consolidation at 6 months after the third ESWT session. (**F**) Maintained union at the 2-year follow-up. *ESWT*, extracorporeal shock wave therapy.

Radiographic callus formation became evident after ESWT and progressed after subsequent sessions. At 6 months after the third ESWT session, substantial consolidation was observed, and union was maintained at the 2-year follow-up. The patient experienced gradual clinical improvement without ESWT-related complications (Fig. 4).

Clinical characteristics and treatment details are summarized in Table I. The follow-up courses were completed from 2019 to 2021 in Case 1, from 2020 to 2023 in Case 2, and from 2021 to 2024 in Case 3.

**Table I:** Summary of the 3 cases.

Case	Age/sex	Risk factors/comorbidity	Fixation-related concern at ESWT	ESWT protocol	Outcome/message
1	49/F	Non-smoker; no alcohol abuse; no diabetes; radial nerve symptoms improved	Nail/screw loosening; no breakage/displacement	0.4 mJ/mm^2^; 3,000 impulses; 4 sessions	Early healing at 2 mo; substantial consolidation within 8 mo; remodeling maintained through 24-mo follow-up; implant removed after union
2	60/M	Non-smoker; no alcohol abuse; controlled diabetes	Limited screw/implant loosening; no catastrophic failure	0.4 mJ/mm^2^; 4,000 impulses; 3 sessions at 2-week intervals	Progressive union by 7-12 mo after the third ESWT session; union maintained at the 2-y follow-up
3	72/M	Non-smoker; no alcohol abuse; no diabetes	More evident screw backing-out; no displacement/breakage	0.4 mJ/mm^2^; 4,000 impulses; 3 sessions at 6- to 8-week intervals	Substantial consolidation at 6 mo after the third ESWT session; union maintained at the 2-y follow-up

*ESWT*, extracorporeal shock wave therapy.

All cases were treated using a piezoelectric-type focused ESWT device under sonographic guidance.

## Discussion

This case report describes 3 patients with humeral shaft delayed union or non-union accompanied by implant loosening or fixation-related concerns who were treated with focused ESWT. All patients demonstrated progressive radiographic healing and ultimately achieved union without revision fixation or bone grafting. In this report, 2-year radiographs represent follow-up confirmation of maintained union rather than the time point at which union first occurred. The clinical value of this report is not the demonstration of union in isolated cases, but the observation that focused ESWT was followed by union in selected cases in which implant loosening or fixation-related mechanical compromise would ordinarily prompt consideration of revision fixation.

Stable mechanical fixation is a fundamental principle in the treatment of fracture delayed union and non-union. The humeral non-union literature emphasizes restoration of stability together with biological augmentation. In a 48-patient humeral shaft aseptic non-union series, plating with supporting allograft strut and bone substitute augmentation achieved full bone healing without major complications.^8^ In distal-third humeral shaft aseptic non-union, plate-and-bone-strut fixation with bone-chip augmentation achieved union in all 26 reported cases.^9^ For proximal humeral aseptic non-union, a 16-patient prospective series used lateral plate fixation combined with medial strut allograft and bone-chip grafting, emphasizing the combined mechanical and biological rationale of treatment.^10^ In intra-articular distal humeral aseptic non-union, double plating with biological or artificial bone graft augmentation has also been reported as an effective revision strategy.^11^ These surgical studies appropriately frame the standard of care: mechanical stability is usually restored by revision fixation, often with autologous, structural, or artificial bone augmentation.

In cases with implant loosening, progressive displacement, or fixation failure, revision fixation with or without bone grafting is generally recommended to restore mechanical stability and biological potential. Therefore, ESWT should not be considered a replacement for adequate mechanical stabilization in clearly unstable non-union. However, the present cases are clinically noteworthy because progressive union was achieved after focused ESWT despite radiographic implant loosening or fixation-related mechanical compromise. These cases may represent a narrow clinical stability window: micro-motion or construct compromise was present, but catastrophic construct failure, progressive displacement, or implant breakage had not yet occurred. Within this window, focused ESWT may help trigger osteogenesis and callus maturation, provided that patients are carefully selected and revision surgery is refused or clinically undesirable. In this context, focused ESWT should be interpreted as a potential biological adjunct or salvage option, not as an alternative standard to revision fixation in unstable humeral non-union.

Previous clinical studies have reported favorable results of ESWT for long-bone non-union. Wang et al^13^ reported shock wave treatment in 72 long-bone non-union cases, including humeral cases. Cacchio et al^2^ compared ESWT with surgery for hypertrophic long-bone non-union in a 126-patient randomized study. Haffner et al^5^ reported encouraging outcomes in 116 tibial fracture non-union patients who were unresponsive to conventional therapy. Willems et al^14^ systematically reviewed ESWT for delayed union and non-union fractures and found promising but heterogeneous evidence. Sansone et al^12^ summarized 23 studies including 1,200 long-bone non-unions, with union after ESWT in 876 cases; hypertrophic non-unions showed higher healing rates than oligotrophic or atrophic cases. Humerus-specific clinical data from Dahm et al^4^ included 236 patients with humeral delayed union or non-union and suggested that focused high-energy ESWT may be considered in well-selected patients, although 6-month consolidation rates varied by anatomic region. Thus, the existing literature supports the biological plausibility and clinical potential of ESWT, but it also underscores that healing rates are variable and that mechanical and biological selection factors remain critical.

Another notable aspect of the present report is that ESWT was applied before catastrophic fixation failure occurred. All 3 cases showed fixation-related concerns, including implant loosening or limited screw/construct backing-out, but none showed implant breakage, progressive displacement, or global loss of fixation requiring immediate revision. Case 1 represented late-stage non-union with substantial nail loosening, Case 2 represented established delayed union with plate-related mechanical compromise, and Case 3 represented early delayed union with more evident interfragmentary screw backing-out. Early adjunctive treatment in Case 3 appeared to be followed by callus progression before gross construct failure developed, whereas late salvage treatment in Case 1 still resulted in eventual union. However, these observations do not prove that ESWT accelerated healing compared with continued observation. Spontaneous late progression cannot be excluded in a three-case report without controls. The rationale for ESWT in these cases was that radiographic progression had stalled and fixation-related concerns were developing, a setting in which revision fixation would ordinarily be discussed. ESWT was therefore used as active biological stimulation under close radiographic monitoring rather than as simple observation.

The number of ESWT sessions was determined pragmatically according to chronicity and perceived mechanical compromise rather than by a rigid protocol. Case 1 received 4 sessions because the non-union was long-standing and accompanied by more pronounced nail loosening, whereas Cases 2 and 3 received the commonly used three-session protocol in our practice. A representative clinical photograph using a volunteer model was added to show the sitting position, arm support with a pillow on the treatment table, ultrasound localization, and focused ESWT applicator positioning over the humeral shaft region. This image is intended to illustrate the treatment setup and is not presented as patient-specific clinical data.

The morphology of non-union is also relevant. The cases in the present report were more consistent with oligotrophic or atrophic biology than with exuberant hypertrophic non-union. ESWT may be more predictable when biology is impaired but residual stability remains, or when hypertrophic non-union exists without severe mechanical failure. Conversely, severe atrophic non-union with a large persistent gap, multiple failed operations, progressive displacement, or marked implant loosening should prompt revision fixation rather than ESWT alone. Recent prognostic factor analysis has similarly identified factors such as atrophic non-union, a larger fracture gap, and previous operations as unfavorable for ESWT success.^15^

Potential disadvantages of ESWT include cost, the need for repeated treatment sessions, discomfort during treatment, local pain, skin erythema, swelling or ecchymosis, and the time required to determine whether callus progression will occur. The most important clinical risk is delayed revision surgery if ESWT is applied to a construct that is already mechanically unsalvageable. Neurovascular structures and the thoracic cavity must be avoided when treating the humerus, and ESWT should not be applied in the presence of active infection or other contraindications. Accordingly, patients should be counseled that revision fixation remains necessary if displacement progresses, implant failure develops, symptoms worsen, or radiographs fail to show healing progression.

The biological effects of ESWT may explain its potential role in fracture healing. Experimental and clinical studies have suggested that ESWT can induce mechanotransduction, promote angiogenesis, increase local growth factor expression, and stimulate osteogenic differentiation.^3^^,^^7^ These effects may reactivate the healing process at a delayed union or non-union site. In the present cases, the proposed role of ESWT was not to restore mechanical stability, but to enhance biological activity at the fracture or non-union interface where some residual construct stability remained. Recent prognostic factor analyses further support the importance of patient selection when ESWT is used for long-bone non-union.^15^

This report has several limitations. First, it includes only 3 cases and lacks a control group. Second, the treatment timing and interval differed among patients. Third, formal functional outcome data, such as visual analog scale pain scores, range of motion measurements, or validated upper-extremity scores, were not available; therefore, clinical improvement was assessed based on symptom improvement documented in the medical records. Fourth, radiographic union was assessed using plain radiographs alone. Computed tomography would have provided a more objective assessment of the fracture gap and bridging bone, but it was not obtained because clinical decision-making and follow-up in these cases were based on serial radiographs and symptoms. Fifth, fixation-related mechanical compromise was assessed radiographically and was not quantified using computed tomography or objective mechanical testing. Sixth, formal bone metabolic status and bone mineral density were not routinely assessed, and a meaningful institutional success rate cannot be estimated from 3 completed reported cases. Finally, ESWT should not be generalized to cases with catastrophic construct failure, progressive displacement, implant breakage, active infection, or an unsafe acoustic pathway. Nevertheless, this report provides clinically relevant observations regarding focused ESWT as a potential adjunctive or salvage option for selected humeral shaft delayed union and non-union cases with implant loosening or fixation-related concerns.

## Conclusion

Focused ESWT may be considered as a non-invasive salvage adjunct in selected patients with humeral shaft delayed union or non-union accompanied by implant loosening or fixation-related mechanical compromise, particularly when revision fixation is refused or clinically undesirable. Successful application appears to depend on meticulous patient selection, specifically cases without catastrophic construct failure, progressive displacement, implant breakage, active infection, or an unsafe acoustic pathway. ESWT should not be considered a replacement for revision fixation in clearly unstable non-union.

## Disclaimers:

Funding: No funding was disclosed by the authors.

Conflicts of interest: The authors, their immediate family, and any research foundation with which they are affiliated have not received any financial payments or other benefits from any commercial entity related to the subject of this article.

Patient consent: Obtained.
